# Methodological and Clinical Concerns in the Study on Postpartum Hypertension Management

**DOI:** 10.1016/j.jacadv.2025.101852

**Published:** 2025-05-31

**Authors:** Takaki Tanamoto, Kyosuke Kamijo, Yusuke Tsutsumi, Tetsuya Kawakita

**Affiliations:** a United States Naval Hospital Yokosuka, Yokosuka, Kanagawa, Japan; b Network for Young Obstetricians and Gynecologists in Japan (NYOG-Japan), Nagano, Japan; c Nagano Prefectural Shinshu Medical Center, Suzaka, Japan; d Scientific Research WorkS Peer Support Group (SRWS-PSG), Osaka, Japan; e National Hospital Organization Mito Medical Center, Ibaraki, Japan; f Kyoto University Graduate School of Medicine, Kyoto, Japan; g Macon & Joan Brock Virginia Health Sciences Eastern Virginia Medical School at Old Dominion University, Norfolk, Virginia, USA; h Center for Maternal and Child Health Equity and Advocacy, Eastern Virginia Medical School, Norfolk, Virginia, USA

We read with interest the recent study by Rosenfeld et al.^1^ However, we have several clinical and methodological concerns about this study.^1^

First, this study's propensity score matching (PSM) lacked key clinical confounders, including perinatal risk factors and the highest systolic/diastolic blood pressures. Smoking, alcohol use, and multifetal gestation, which influence blood pressure during pregnancy,^2^^,^^3^ potentially affect both primary and secondary outcomes. Additionally, the authors did not report standardized mean differences before and after PSM, which are essential for assessing balance.^4^

Second, we noted inconsistencies in the methodological approach of their outcomes, causing potential differences in the analyzed populations. Both risk difference (RD) and OR were obtained using PSM-adjusted analysis. However, while the RD was calculated without considering matched pairs, the OR incorporated this adjustment. Additionally, the method for calculating the “doubly robust (DR) OR” was not well documented. These discrepancies make it difficult to determine which analytic method represents the primary analysis. Furthermore, why different analytical approaches were applied to RD and OR remains unclear. We seek clarification on the methodology used for DR OR. Regarding the DR OR, the authors suggested that additional adjustment for the same covariates used in the PSM was applied again after PSM. If so, this raises concerns about potential over adjustment and the introduction of biased estimations.

Third, the study offers no justification for employing a 1:3 matching ratio in PSM. Using 1:3 matching instead of 1:1 can increase bias, particularly if the additional matched controls are less comparable to the treatment group.^5^

By addressing these concerns, the authors can substantially strengthen the validity and rigor of their study, thereby enhancing its value to both clinical practitioners and researchers.
